# Regulatory and metabolic control of microbial biosynthetic gene clusters

**DOI:** 10.1038/s42003-026-10623-z

**Published:** 2026-07-17

**Authors:** Idris Matine, Frederick Clasen, Fernando Garcia Guevara, Miao Guo, Saeed Shoaie

**Affiliations:** 1 Centre for Host-Microbiome Interactions, Faculty of Dentistry, Oral & Craniofacial Sciences, King’s College London, London, UK; 2 Department of Engineering, Faculty of Natural, Mathematical & Engineering Sciences, King’s College London, London, UK; 3 Quantitative Systems Biology, Faculty of Medicine, Biruni University, Istanbul, Turkey

**Keywords:** Biosynthesis, Computational biology and bioinformatics, Microbiology

## Abstract

Microbial biosynthetic gene clusters (BGCs) encode diverse bioactive molecules but remain mostly silent under lab conditions, reflecting a balance between regulatory control and the metabolic cost of secondary metabolite production. While activation strategies exist, the yields stay low because switching on a cluster doesn’t guarantee the host can sustain biosynthesis. This review covers the regulatory architecture and metabolic constraints, including precursor availability, energy status, and nutrient sensing, that shape BGC output. We argue that coupling regulatory network models with genome-scale metabolic models offers a powerful framework for unlocking silent BGCs and realising the full biosynthetic potential within microbial genomes.

## Abstract (web summary)

This review summarises multilayered regulatory and metabolic systems governing silent biosynthetic gene clusters in microbes, and how coupling regulatory network models with genome-scale metabolic models can unlock their biosynthetic potential

## Introduction

For millennia, we have turned to nature for medicinal solutions, treating illnesses like infections and pain with remedies derived from plants and other natural sources. Over the last two centuries, scientific advances have shifted the focus from using mixtures to isolating and analysing the specific compounds responsible for therapeutic effects^1,2^. Advances in high-throughput data generation and whole-genome sequencing, together with culturomics, marked a turning point in the investigation of microbial communities for a wider array of bioactive substances^2^. Moreover, shotgun metagenomics enables researchers to study microbial communities directly in their natural environments, especially for more challenging species to isolate and grow independently^3–5^. These approaches reveal not only the diversity and functions of these ecosystems but also their complex relationships with hosts^6–8^. The generation of large-scale metagenomic datasets has enabled the discovery of novel genes and, through rigorous functional annotation, has revealed the enzymatic activities present across diverse microbiome habitats. This approach has uncovered an untapped reservoir of millions of previously unexplored genes and biosynthetic pathways^9–12^.

Natural products from both traditional plant sources and newly identified microbes continue to be invaluable, providing insights into biosynthetic pathways and forming the basis for many essential drugs^13,14^. Approximately half of all approved small-molecule drugs and crop protection agents are either natural products or their direct derivatives^15,16^. A major class of natural products is secondary metabolites (SMs), organic compounds synthesised by living organisms that do not play a direct role in their normal growth, development or reproduction^17,18^. Microbial SMs remain one of our richest sources for new therapeutics and are increasingly explored as eco-friendly alternatives in industries like fuels and cosmetics^19–21^. The genetic blueprints for these metabolites are encoded in colocalised sets of genes known as biosynthetic gene clusters (BGCs)^6,22^. These clusters typically contain all the necessary components for production: core enzymes to build the molecular backbone, tailoring enzymes for chemical modification, regulators to control expression, and transporters for export^23–25^. The modular nature of BGCs underpins the remarkable chemical diversity of SMs and remains a key focus for their discovery^17,24,26^.

Despite their immense potential, interest in SM discovery from pharmaceutical companies has declined over the past few decades^27^. These challenges span the entire development pipeline, from heterologous expression of novel genes in biotechnology model organisms, pathway engineering, elucidating molecular regulation, and the low hit-rates of traditional screening methods to the significant difficulties in establishing reliable, large-scale bioprocess manufacturing, all of which are expensive, time-consuming, and have yielded diminishing returns^27,28^. With the rapid advancement of genome sequencing technologies and BGC prediction tools, it is clear that the biosynthetic potential encoded within microbial genomes is far greater than previously anticipated^6–8^. However, the vast majority of these BGCs remain transcriptionally inactive, or “silent,” under standard laboratory conditions^29,30^. A variety of strategies have been developed to activate these clusters, ranging from untargeted perturbations that globally reshape cellular physiology to precise genetic interventions made possible by emerging genome engineering tools^20,31,32^. However, even when activation is achieved, production levels remain low relative to biotechnological objectives; the yields sufficient for ecological functions such as competitor inhibition are frequently inadequate for industrial applications, highlighting the persistent metabolic and regulatory constraints that limit scalable SM biosynthesis^33,34^.

The transcriptional inactivity may stem from long-term evolutionary adaptations, driven by specific metabolic niche requirements and reinforced by tight regulatory control. To achieve ecological success, microbes rely on primary metabolism, a core set of reactions that generate the energy and building blocks required for growth, repair and replication^18,35,36^. The production of SMs taps into this same, finite pool of resources, creating an intense metabolic competition, as the synthesis of complex SMs imposes significant energetic and precursor burden^37,38^. This competition forces a fundamental trade-off between proliferation and the production of specialised metabolites, which primarily function in ecological competition^30,37^. This high-stakes metabolic trade-off may be why biosynthetic pathways are so tightly regulated by a hierarchy of regulatory elements, ensuring they are only expressed when the ecological benefit outweighs the metabolic cost^38–40^.

To address the challenge of silent BGCs, a comprehensive systems-level understanding of their control mechanisms is required. Many regulatory constraints (transcriptional, epigenetic, and post-transcriptional) limit scalable SM biosynthesis^33,39^, highlighting a critical challenge: many BGCs depend on native regulatory and metabolic cues that are absent in model hosts, so heterologous activation often fails to achieve robust SM production. Therefore, there is a critical need to deepen our understanding of BGC transcriptional regulation within native, non-model microbial hosts. While model biotechnology organisms offer tractable genetics and standardised engineering tools, their capacity to faithfully replicate required conditions for diverse SM production is inherently limited^31,41^. Many pathways rely on host-specific transcription factors (TFs), signalling networks, chromosomal organisation, and metabolic cues that are absent or fundamentally altered in heterologous systems. As a result, transferring BGCs into model hosts often leads to suboptimal expression, reduced stability, poor scalability, and unpredictable biomanufacturing performance. Investigating transcriptional regulation directly in native producers is therefore essential for uncovering the full spectrum of regulatory logic, enabling more accurate pathway activation strategies, and ultimately expanding the accessible chemical space of microbial SMs^42^.

**Fig. 1:**
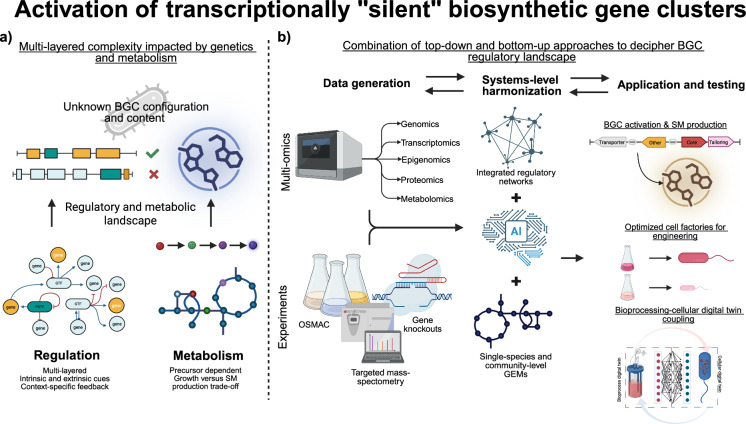
Integrated systems biology framework for deciphering and activating silent biosynthetic gene clusters. Illustration of a workflow for a deeper understanding of biosynthetic gene cluster (BGC) silence to drive rational activation and yield optimisation. **a** depicts the biological challenge, where BGC expression is governed by two interconnected constraints: multilayered transcriptional regulation by pathway-specific (PSTF) and global transcription factors (GTF), and the metabolic burden imposed by specialised metabolite synthesis competing with growth. **b** depicts how multi-omics data (genomics, transcriptomics, epigenomics, proteomics, metabolomics) and experimental perturbations, including one-strain-many-compounds (OSMAC) culture condition variation, gene knockouts, and targeted mass spectrometry, are integrated through AI-assisted frameworks coupling regulatory network models with genome-scale metabolic models (GEMs) to guide targeted BGC activation, metabolite discovery, and engineering of optimised microbial cell factories. Ultimately, bioprocessing-cellular digital twin models will enable rapid virtual experimentation, accelerating the discovery and optimisation pipeline. Created in BioRender. Matine, I. (2026) https://BioRender.com/y8supk6.

Despite advances in BGC discovery and activation, the interface between regulatory control and cellular metabolism is often studied in isolation, and an integrated perspective is still lacking. This review presents an integrated overview of the two fundamental components that govern BGC activation: regulation and metabolism. The multilayered regulatory architecture is examined, followed by an analysis of how these regulatory processes interface with cellular metabolic state, including the roles of nutrient availability, energetic constraints, and the economic trade-offs inherent to specialised metabolite production. Current activation strategies and their limitations are summarised, and emerging systems biology and computational modelling approaches are highlighted as promising frameworks for unifying regulatory and metabolic perspectives to enable the rational activation of silent gene clusters and the achievement of higher, more robust production yields (Fig. 1).

## Multi-layered regulation of biosynthetic gene cluster expression

The most direct routes to activating silent BGCs ultimately rely on manipulating the regulatory systems that control their expression. Although a wide range of experimental techniques has been developed to trigger specialised metabolite production, the effectiveness of any method depends on the underlying regulatory architecture that dictates whether a cluster remains silent or becomes active^20,31,42^.

The silence of BGCs is enforced by a multi-layered regulatory system, a complex “code” that must be deciphered to unlock their biosynthetic potential^43,44^. This code is written at multiple levels, from the direct on/off switches of TFs to the global accessibility controlled by chromatin structure in eukaryotes, and the fine-tuning provided by post-transcriptional modifications. Efforts to crack this code, whether through genetic manipulation in the native host or via heterologous expression in a more tractable organism, depend on understanding the interconnected layers^44^. However, the complexity of this system often makes these approaches challenging, requiring extensive refactoring and optimisation to achieve effective results^44,45^.

### Transcriptional regulation - pathway and global transcription factors

BGCs are regulated through hierarchical networks that integrate multiple layers of control, allowing cells to coordinate SM production with physiological and environmental cues. At the top of this hierarchy are global TFs (GTFs), which sense broad metabolic or environmental signals and modulate the overall cellular state. Downstream are pathway-specific TFs (PSTFs), often encoded within or adjacent to the cluster, which act as dedicated switches to coordinate expression of all biosynthetic genes. Additional regulatory layers, including local repressors, small-molecule ligands, and alternative sigma factors, fine-tune pathway activation, while feedback loops ensure robust and sustained transcription once initiated. This layered architecture allows BGCs to integrate global cues with pathway-specific signals, ensuring metabolite production occurs in a context-dependent and tightly controlled manner^39^.

**Fig. 2:**
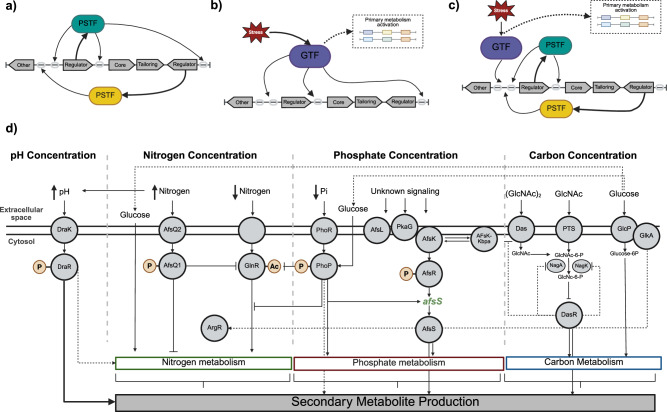
Multi-layered transcriptional regulation of biosynthetic gene clusters and integration of environmental cues. **a** Pathway-specific regulation: biosynthetic gene clusters (BGCs) often encode one or more pathway-specific transcription factors (PSTFs) that provide direct, fine-tuned control of biosynthetic genes, forming feedback loops and cross-regulating multiple operons within the cluster. **b** Direct global regulation: smaller BGCs may lack a dedicated PSTF and are instead directly regulated by a global transcription factor (GTF) in response to broad physiological or stress signals, coordinating expression of both secondary and primary metabolism genes. **c** Hierarchical regulation: a GTF senses external cues and regulates PSTFs, which in turn activate the remaining genes within the cluster. **d** Integration of environmental cues in Streptomyces: key nutrient- and stress-sensing regulatory networks, including pH (DraR-DraK), nitrogen (AfsQ1-AfsQ2, GlnR), phosphate (PhoR-PhoP, AfsR), and carbon (DasR), converge to modulate secondary metabolism. Solid arrows indicate direct regulation, dashed arrows indicate indirect influence, and inhibitory interactions are shown with blunt-ended lines. Phosphorylation and acetylation are indicated by ‘P’ and ‘Ac’ respectively. Created in BioRender. Matine, I. (2026) https://BioRender.com/y8supk6.

A foundational example is ActII-ORF4, a SARP-family transcriptional activator that governs the biosynthesis of actinorhodin (ACT), a well-known blue-pigmented polyketide antibiotic in *Streptomyces coelicolor*^46,47^. PSTFs commonly coordinate expression across the entire pathway by binding multiple promoter regions within the cluster, ensuring that all biosynthetic genes are activated in a synchronised manner (Fig. 2a). This principle is illustrated by StrR, the regulator of the streptomycin BGC in *S. griseus*, which directly activates numerous genes required for antibiotic production^48^.

The roles of these local TFs are not always straightforward and can be evolutionarily dynamic. XanC, a PSTF in fungi, activates the xanthocillin BGC in *Aspergillus fumigatus*. In a closely related fungus *Penicillium expansum*, the XanC homologue has been evolutionarily “rewired” and no longer controls xanthocillin but instead activates the entirely different citrinin BGC^40,49^. This highlights that the function of a PSTF is context-dependent and can diverge even between related species, which is important to consider when building regulatory network models.

PSTFs rarely operate in isolation. Their activity is typically the final output of a broader regulatory cascade, raising an essential question: what regulates the regulators? The expression of a PSTF is itself a critical control point, shaped by multiple regulatory layers that allow the cell to integrate diverse physiological and environmental cues^39^. One common mechanism involves local repressors, often TetR-family proteins, which bind to the PSTF promoter and physically block transcription. This repression is relieved only when the repressor binds a specific small-molecule ligand, triggering its release and allowing transcription to proceed^50,51^. A well-studied example is the γ-butyrolactone signalling system in *Streptomyces coelicolor*, where accumulation of the signal molecule lifts repression of the PSTF gene and activates the associated cryptic BGC^36,52,53^. In this system, the signalling molecule SCB1 accumulates in a population-density–dependent manner. Its cognate receptor, ScbR, is a TetR-family repressor that binds directly to the promoter of *kasO* (also known as *cpkO*), the PSTF for a cryptic polyketide BGC. In vitro DNA-binding assays, including DNase I footprinting, have confirmed that ScbR physically occupies the *kasO* promoter, blocking transcription. When SCB1 reaches a critical threshold concentration, it binds to ScbR and induces a conformational change that releases the repressor from the DNA. This de-repression permits *kasO* transcription, and the resulting PSTF then activates the entire cluster^53^.

Beyond repressor–ligand systems, BGC activation can also be driven by alternative sigma factors that become active only under defined conditions such as stress or intercellular communication^50,54,55^. This mechanism is particularly common in Actinomycetes, which encode numerous Extracytoplasmic Function sigma factors. Although many of these sigma factors govern general stress responses, several are located within BGCs and function as dedicated, pathway-specific regulators^50^. For example, AntA is an orphan extracytoplasmic function sigma factor conserved across antimycin BGCs in many *Streptomyces* species. Once active, AntA directly initiates transcription of key operons required for antimycin production^54^.

The most prominent mode of BGC regulation occurs through GTFs. BGCs have a wide range of architectures and, in some cases, can lack PSTFs and are instead fully controlled by GTFs that bind directly to biosynthetic gene promoters^39,56^ (Fig. 2b). More commonly, however, is hierarchical regulation where GTFs act as the primary sensors of environmental or metabolic cues and induce expression of PSTFs that in turn activate the remaining genes^39^ (Fig. 2c). Many PSTFs also enhance transcription of their own gene, creating a positive feedback loop that stabilises and amplifies pathway activation once initiated^39,57^.

GTFs respond to environmental and metabolic conditions such as phosphate, nitrogen, and carbon availability^58,59^ and can override or reinforce pathway-specific activation, ultimately determining whether a BGC is turned on. In the case of *Streptomyces pactum*, there is a layered regulation of the pactamycin BGC. Although the cluster encodes two pathway-specific activators, PtmE and PtmF, their activity is suppressed by the global phosphate-response system PhoR–PhoP^60^. High levels of inorganic phosphate activate PhoP, which completely blocks pactamycin production. Deleting *phoP* removes this inhibition and restores biosynthesis even under phosphate-rich conditions. PhoR–PhoP is one of the best-studied global regulatory systems in *Streptomyces*, linking external phosphate availability to major shifts in cellular physiology^28,42,61^. Under phosphate-limiting conditions, PhoR activates PhoP, which induces genes for phosphate acquisition while simultaneously relieving repression of multiple secondary-metabolite pathways^58,62^. This regulatory shift rebalances cellular priorities from rapid growth toward stress adaptation and specialised metabolism. PhoP activity also interfaces with other global regulators, including the AfsKRS system, creating a broader network that coordinates antibiotic biosynthesis across the genome^63,64^.

GlnR and AfsQ1 are key examples of regulatory systems that couple changes in nitrogen availability to biosynthetic output^65–67^. Under nitrogen-limiting or nitrogen-replete conditions, the activity and DNA-binding behaviour of these regulators is altered, enabling cells to reprogramme metabolic and secondary metabolic pathways accordingly. In *Streptomyces venezuelae*, GlnR binds to at least 36 sites across the genome, linking nitrogen sensing to secondary metabolism, catabolic processes, and transport functions^68^. The regulatory outcome of GlnR is species-specific. In *S. coelicolor*, deletion of *glnR* increases ACT production but reduces undecylprodigiosin levels by repressing *actII-ORF4* (the ACT activator) while activating *redZ* (the undecylprodigiosin activator). In *S. avermitilis*, GlnR promotes avermectin biosynthesis via *aveR* but represses oligomycin production through *olmRI/RII*^65^. Together, these examples show how nitrogen-dependent modulation of GlnR activity enables differential coordination of multiple BGCs in response to nitrogen availability.

Carbon catabolite repression introduces an additional layer of global regulation. *In S. coelicolor*, glucose suppresses expression of the *act* and *red* clusters but activates the coelimycin P1^69^. This demonstrates that carbon control is not simple repression but a dynamic reconfiguration of secondary metabolism. The regulator DasR plays a central role in this process by responding to N-acetylglucosamine (GlcNAc), a signal of microbial activity^70^. Without GlcNAc, DasR represses *actII-ORF4* and *redD*. When GlcNAc is present, it is converted to glucosamine-6-phosphate (GlcN-6-P), which inactivates DasR and lifts repression.

These global networks do not act independently. Although the PhoR–PhoP system mainly controls phosphate responses, it also interacts with other nutrient-sensing systems. PhoP binds to the promoter of *glnR*, repressing its activation and influencing nitrogen metabolism^71^. Furthermore, the phosphate-binding protein *PstS*, part of the phosphate transport system, has been linked to sensing environmental signals that affect carbon metabolism^72^. Such interactions show that global regulators communicate with each other to fine-tune metabolic balance. The combined phosphate, nitrogen, and carbon networks form complex transcriptional circuits that tightly control secondary metabolism (Fig. 2d), as described in several recent reviews^58,59,61^.

Exploiting this regulatory hierarchy has proven effective in activating silent clusters^73–75^. For instance, the overexpression of *actII-ORF4* is a well-established method to drive robust ACT biosynthesis, even during exponential growth, where the cluster is normally silent^76^. However, this approach is constrained by the difficulty of identifying the correct activating regulator for a given BGC, whether it is a PSTF encoded within or adjacent to the cluster or the upstream GTF that governs its expression^39^. Genome-wide studies have increasingly highlighted the pivotal role of global regulators, shifting the paradigm from a cluster-centric view toward a systems-level understanding of secondary metabolism^23^. A holistic understanding of transcriptional architecture and deciphering how local and global signals are integrated to flip the “on/off” switch of a BGC is still lacking in the field^77^.

### Epigenetic and post-transcriptional regulation

Physical accessibility to DNA often acts as a master switch for transcriptional activation. When the regulatory region of a BGC is tightly compacted, the cluster is likely to remain silent, even in the abundant presence of activators^31^.

In bacteria, chromosome organisation is managed by nucleoid-associated proteins, small polypeptides that alter DNA topology via bridging, wrapping, and bending^78,79^. These proteins are crucial for how bacteria cope with environmental stress, governing large sets of genes, including those for specialised metabolism^74,75^. Among the best-characterised examples in Streptomyces is Lsr2, a master silencer that preferentially targets AT-rich DNA regions, a feature of many polyketide synthase (PKS) and non-ribosomal peptide synthetase (NRPS) BGCs^80^. Functional disruption of *lsr2* leads to the broad de-repression of cryptic SM loci^73,80^.

In fungi, many BGCs reside within heterochromatic regions of the genome, where transcription is typically repressed^81^. Increased histone acetylation typically activates, whereas histone methylation silences BGC expression^82,83^. However, this distinction is an oversimplification, and most fungal BGCs remain silent despite efforts to manipulate these general marks. For example, in *Fusarium graminearum*, activation of the Bikaverin BGC requires not only histone acetylation but also the removal of a specific repressive mark, H3K27 methylation, by a dedicated demethylase enzyme^84–87^. This illustrates a more complicated “histone code,” where the interplay between different modifications and the global regulators that interpret them, such as the chromatin-remodelling factor LaeA, ultimately fine-tunes BGC expression^88^. Several excellent reviews provide comprehensive overviews of fungal chromatin biology and its role in SM regulation^82,83,85^.

Post-transcriptional mechanisms could provide an additional layer of control over BGC expression, offering the potential for rapid regulatory responses without requiring changes to transcriptional activity^89–91^. This is often mediated by small non-coding RNAs, which can target mRNAs for degradation or block their translation^92^, and by riboswitches, structured mRNA elements that directly bind metabolites to halt their own synthesis in a direct feedback loop^93,94^. While these mechanisms are well-characterised for individual genes, their specific roles in the coordinated regulation of entire, often polycistronic, BGCs are not well studied and represent a significant knowledge gap^95^. A major unresolved question is how a single post-transcriptional regulatory event acting on a polycistronic transcript influences the stoichiometric balance of the multiple enzymes encoded within a BGC. Elucidating such mechanisms represents a largely unexplored frontier in achieving a comprehensive view of BGC regulatory architectures.

Regulatory elements do not act in isolation but function as interconnected components of complex regulatory networks^96–98^, motivating a shift from studying individual regulators toward systems-level models of control^23,99^. While most current efforts focus on TF–centric networks, broader frameworks in other fields increasingly integrate chromatin accessibility and additional regulatory layers to achieve a more complete view of gene regulation^99–104^. Even such integrated regulatory models, however, are not sufficient to explain BGC output, which is ultimately constrained by metabolic capacity, including precursor availability, cofactor and energy status, and flux through central metabolism^105^. Incorporating these metabolic dimensions is therefore essential for a true systems-level understanding of secondary metabolism.

## Metabolism and resource allocation in secondary metabolite production

Regulation determines when a BGC should be activated, but metabolism determines whether the cell can afford to execute that decision^59,106,107^. Therefore, metabolic state is central to understanding the logic and constraints of BGC expression^106^. Fundamentally, the decision to activate a BGC is an economic one, shaped by both the external environment and the cell’s internal biochemical state. The success of one strain–many compounds strategies (Table 1) illustrates how environmental variation reshapes cellular metabolism in ways that directly influence BGC expression^108,109^. In this context, precursor availability is critical because SMs draw on the same building blocks required for primary metabolism^110–112^. SM biosynthesis is also energetically demanding, relying on multiple reductive and oxidative steps that consume substantial amounts of ATP and NADPH^113,114^. This direct competition for finite cellular resources underlies the evolution of the regulatory systems described earlier in this review^106,115^.

**Table 1:** Summary of strategies for activating BGCs. Adapted and modified from ref. ^31^

Approach	Strategy	Description	Example	Advantages	Limitation	Ref
Pleiotropic (Global) Methods						
**Culture Based**	**Culture Variation (One Strain-Many Compounds)**	Altering media composition, nutrients, pH, temperature, or aeration to shift regulatory and metabolic states and reveal otherwise silent pathways.	Supplementing solid rice medium with fruit or vegetable juice when cultivating *Fusarium tricinctum* induced production of new metabolites fusarielin J, K, and L, and increased fusarielin J accumulation.	High throughput and easy to implement. Often identifies unexpected metabolic triggers.	Outcomes are unpredictable and often produce complex metabolite mixtures.	^166^
	**Co-cultivation**	Growing two or more species together to mimic competition or symbiosis and elicit interaction-dependent BGCs.	Co-culturing *Aspergillus nidulans* with soil-derived *Streptomyces* species activated a normally silent PKS gene cluster, inducing production of orsellinic acid, lecanoric acid, and the cathepsin K inhibitors F-9775A and F-9775B.	Reflects natural ecology and reveals metabolites that depend on microbial interactions.	Low throughput and difficult to scale, especially with incompatible growth rates.	^167^
**Genetic Manipulation**	**Ribosome/ RNAP Engineering**	Introducing mutations to core transcription and translation machinery	A point mutation in the *rpsL* gene of *Streptomyces lividans* (Lys88→Glu), selected via streptomycin resistance, activated production of the normally silent antibiotic ACT.	Requires no prior regulatory knowledge and works across diverse hosts.	Effects are unpredictable and can impair growth. Some mutations are unstable.	^168^
	**Global Regulator Manipulation**	Overexpressing a global activator or deleting a global repressor to rewire high-level control of secondary metabolism.	Overexpression of the global regulator Talae1 in the endophytic fungus *Trichodermaafroharzianum* led to the production of two novel polyketides that were not produced in the wild-type strain.	Can activate numerous BGCs at once and often produces strong transcriptional responses.	Pleiotropic effects can be harmful and often result in complex metabolite mixtures.	^169^
	**Epigenetic Perturbation**	Using chromatin modifiers such as HDAC inhibitors, DNA methylation inhibitors or deletion of chromatin regulators to switch heterochromatic BGCs into active states.	Deletion of *hdaA* in *Aspergillus nidulans* activated two telomere-proximal BGCs, increasing production of the corresponding toxin and antibiotic, while telomere-distal clusters remained unaffected. Treatment of other fungi with HDAC inhibitors similarly induced overproduction of several metabolites.	Works well on deeply silenced clusters and is straightforward to apply.	Can cause widespread off-target transcriptional changes. Chemical inhibitors can be toxic or stress-inducing.	^170^
**Pathway Specific (Targeted) Methods**						
**Native Host Manipulation**	**Activator/ Repressor Manipulation**	Inducing a known pathway activator using a controllable promoter or deleting a known pathway repressor to relieve transcriptional repression.	Ectopic expression of the pathway-specific regulator in *Aspergillus nidulans* activated a silent PKS-NRPS hybrid gene cluster, leading to transcription of its target genes and the production of two new cytotoxic metabolites, aspyridones A and B.	Highly precise with predictable outcomes and clean metabolic profiles.	Requires prior knowledge of the regulatory genes within the BGC.	^171^
	**Reporter-Guided Screening**	Fusing a BGC promoter to a reporter gene and screening mutagenised populations to identify mutants in which the promoter becomes active.	Reporter guided screening was used in *Streptomyces venezuelae* to reactivate the jadomycin BGC and in *Streptomyces sp*. PGA64 to activate a previously silent *pga* cluster, resulting in the discovery of new anthraquinone aminoglycosides gaudimycin D and E.	Identifies novel regulatory mutations without needing prior information.	Labour intensive and requires appropriate genetic tools.	^172^
	**BGC Refactoring**	Replacing native promoters with well-characterised synthetic promoters to activate the BGC.	In *Streptomyces albus J1074*, insertion of strong promoters activated the production of indigoidine and 6-epi-alteramides A and B.	Provides complete and changeable expression control.	Technically demanding with extensive cloning requirements.	^173^
	**Phage-based genome refactoring**	Using bacteriophage-derived recombinases or phage regulatory elements (promoters, terminators, integrases, sigma-like factors) to rewrite BGC regulatory architecture.	Red-like recombinases from *Burkholderiales* DSM 7029 were used for in situ promoter insertion, activating two silent NRPS/PKS clusters and enabling discovery of glidopeptins and rhizomides.	High precision and effective in non-model hosts. Orthogonal parts avoid host repression and can activate deeply silent clusters	Requires efficient delivery systems and can be species-specific. Large-scale rewiring is labour intensive.	^174,175^
**Heterologous Expression**	**Chassis-Based Production**	Cloning and expressing BGCs in genetically tractable hosts. Technologies such as CRAGE enable rapid integration into a wide range of chassis organisms.	Nine BGCs from *Photorhabdus luminescens* (three known, six previously silent NRPS/PKS-NRPS hybrids) were integrated into 25 γ-Proteobacteria species. Six BGCs were successfully activated, producing 22 natural products, with higher yields in hosts closely related to the donor strain.	Accesses metabolites from unculturable organisms and benefits from optimised host metabolism.	Large BGCs are difficult to clone and transfer, precursor supply may not match the pathway’s needs, and the host’s transcriptional or enzymatic machinery can differ from the native organism,	^176^
**Cell-Free Systems**	In Vitro **Expression (CFE)**	Cell-free systems reconstruct transcription and translation outside the cell, enabling rapid expression and testing of BGCs in crude lysates or defined PURE systems. These platforms allow fast prototyping without the constraints of cellular growth or regulation.	NRPS enzymes BpsA (from *Streptomyces lavendulae*) and KJ12ABC (from *Xenorhabdus KJ12.1*) were expressed in vitro, producing indigoidine and rhabdopeptides, respectively. Post-translational modifications and enzymatic activity were confirmed, demonstrating functional metabolite synthesis.	Extremely fast and not limited by cell viability. Highly suitable for automation.	Still developing and not yet optimal for very large NRPS or PKS enzymes. PURE systems are costly.	^177^

This competition imposes an inherent trade-off between growth and secondary metabolism, whereby investment in one process necessarily limits the other^116,117^. In nutrient-rich environments, cells prioritise rapid proliferation, and carbon sources such as glucose suppress the transcription of many BGCs^69,118^. As nutrients decline, cellular priorities shift. In *Streptomyces*, phosphate limitation sensed by the PhoR–PhoP two-component system initiates a coordinated reprogramming that downregulates growth-associated pathways while upregulating antibiotic BGCs, diverting resources toward specialised metabolism^62,119^. Comparable nutrient-responsive switches occur in filamentous fungi, where shifts in nitrogen availability can activate otherwise silent SM gene clusters in *Aspergillus nidulans*^120^. Even when BGCs are transcriptionally active, biosynthetic output remains constrained by metabolic capacity, as demonstrated by metabolic engineering studies in *Streptomyces*, where overexpression of acetyl-CoA carboxylase increases malonyl-CoA availability and enhances polyketide production such as ACT^59,113,121^. Similar precursor constraints apply to other malonyl-CoA–derived polyketides, including lovastatin biosynthesis in *Aspergillus terreus*^122,123^.

Competition for precursor metabolites is not limited to central carbon metabolism. For example, the immunosuppressant FK506 (tacrolimus) relies on the unusual starter unit (4 R,5 R)-4,5-dihydroxycyclohex-1-enecarboxylic acid that is derived from chorismate, a key intermediate of the shikimate pathway^124^. Because chorismate also feeds the biosynthesis of essential aromatic amino acids, FK506 production directly competes with primary metabolism^125^. Overexpression of enzymes in the shikimate pathway can relieve this bottleneck and boost FK506 yield^126,127^. A similar interplay is seen in clavulanic acid biosynthesis *in Streptomyces clavuligerus*, which draws precursors from both the arginine pathway and glyceraldehyde-3-phosphate^121,128^. Glycolysis disruption (e.g., disruption of *gap1*) redirects carbon toward clavulanate, enhancing production^121^.

**Fig. 3:**
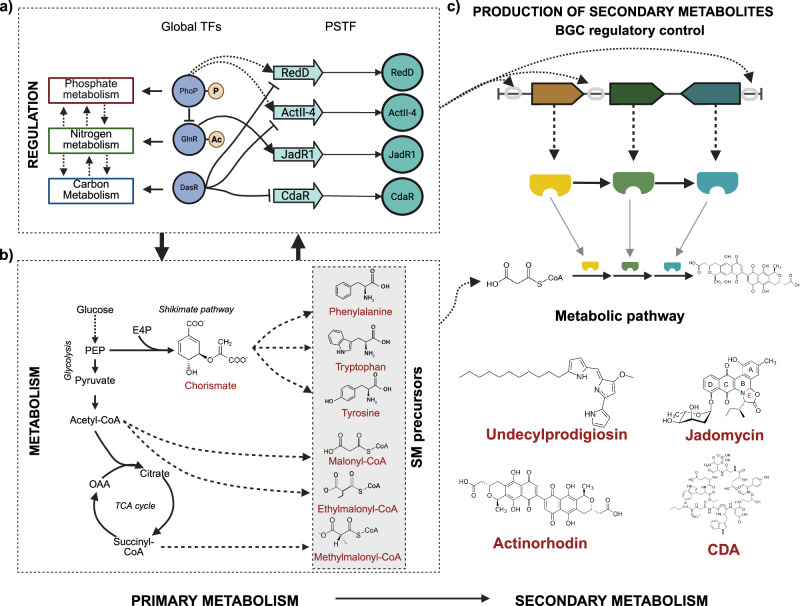
Integrated regulatory and metabolic networks controlling secondary metabolism. Schematic illustrating the interplay between primary metabolism, **a** and **b**, and secondary metabolism, **c**, across three functional layers: regulation, metabolism, and production. Solid arrows indicate activation, T-bars indicate repression, and dotted lines represent multi-step or indirect influences. **a** Regulation layer: hierarchical regulatory cascades link environmental sensing to biosynthetic gene cluster (BGC) activation. Global transcription factors (GTFs) such as PhoP, GlnR, and DasR monitor cellular nutritional status (phosphate, nitrogen, and carbon) and regulate pathway-specific transcription factors (PSTFs), including RedD, ActII-4, JadR1, and CdaR, which control BGC expression within the secondary metabolism domain. **b** Metabolism layer: core metabolic pathways, including glycolysis, the TCA cycle, and the shikimate pathway, generate precursor metabolites such as malonyl-CoA, chorismate, and ethylmalonyl-CoA that fuel secondary metabolite biosynthesis. **c** Production layer: activated PSTFs induce expression of BGC core genes, whose encoded enzymes utilise the supplied precursors to produce structurally diverse secondary metabolites, including undecylprodigiosin, actinorhodin, jadomycin, and calcium-dependent antibiotic (CDA). Created in BioRender. Matine, I. (2026) https://BioRender.com/y8supk6.

This trade-off becomes particularly evident in biotechnology and metabolic engineering. Expressing large gene clusters into hosts such as engineered strains of *E. coli* optimised for rapid growth can drain precursors, cofactors, and energy^117,129^. The conflict between rapid growth and specialised metabolism is therefore not merely theoretical but represents a fundamental challenge in developing stable, high-yielding production strains^41,117^. While many studies have increased SM production by engineering host cells to boost precursor availability^130–132^, these brute-force approaches often yield diminishing returns and can destabilise host physiology. Increasing precursor abundance does not automatically translate into higher production because secondary metabolism operates within a broader physiological context. What ultimately limits output is not a single bottleneck but the interplay between metabolic fluxes, energy availability, and regulatory signals that collectively determine whether a cell can sustain the demands of a BGC (Fig. 3).

Environmental inputs shape GTF activity, which feeds into primary metabolism and can directly or indirectly activate SM pathways. This may occur through the direct induction of BGC promoters or via modulation of PSTFs that govern the expression of pathway enzymes. These enzymes assemble building blocks into the core scaffold before tailoring reactions generate the final product^39^. The system is highly organised yet deeply interdependent. Precursor supply, regulatory cues, and enzymatic activities are tightly coupled, but an often-overlooked dimension of this burden is the translational cost of producing the biosynthetic machinery itself. BGCs frequently encode large enzymes such as PKS and NRPS, whose synthesis places substantial demands on the ribosomal pool, amino acid availability, and GTP consumption^117,133^. Furthermore, the availability of rare tRNAs, such as the bldA-encoded leucyl-tRNA required for UUA codon translation in many BGC regulatory genes, adds a further layer of translational control that can restrict biosynthetic output independently of transcriptional activation^46^. Together, these translational constraints mean that even when precursors are sufficient and regulatory barriers are lifted, the cell must still dedicate significant translational capacity to produce functional biosynthetic enzymes, making SM production a finely balanced process. These complexities highlight the need for systems-level metabolic modelling and engineering frameworks capable of predicting host bottlenecks, optimising flux distributions, and designing production strains that remain robust over evolutionary timescales^33,117,134^.

A deeper understanding of how metabolism and regulatory networks intersect provides insight into why many BGCs remain silent under laboratory conditions. This knowledge also lays the groundwork for rational approaches to activate these pathways, motivating the development of targeted strategies to unlock the full biosynthetic potential of microorganisms^31^.

## Strategies for activating biosynthetic gene clusters

Several strategies have been established to activate silent BGCs by modifying cellular conditions or directly manipulating their regulatory architecture (Table 1). Broadly, these fall into two categories: pleiotropic (global) methods, which act on high-level regulators to reshape the physiological state of the cell, and pathway-specific (targeted) methods, which intervene directly at the level of the BGC or its dedicated regulators.

Pleiotropic activation strategies operate by altering the broader regulatory landscape of the cell. Changes in growth environment such as nutrient limitation, phosphate stress, or nitrogen fluctuations modulate global regulators like PhoP, GlnR, or DasR, which in turn influence large sets of BGCs simultaneously^59,135^. Co-culturing with other microorganisms can further activate BGCs by inducing interspecies signalling pathways^136^, while precursor supplementation can shift metabolic fluxes in ways that favour SM synthesis^137–139^. Random and transposon mutagenesis also fall into this category, as disrupting repressors, competing pathways, or global control elements can lift transcriptional blocks and reveal otherwise silent chemistry^109,140^. Reporter-based and MS-guided screening methods further facilitate the identification of mutants with activated BGC expression, enabling higher-throughput screening^140,141^. Classical random mutagenesis and screening programmes have historically achieved remarkable gains in SM yields, most notably in the iterative strain improvement of penicillin-producing fungi that transformed industrial antibiotic production^142,143^. However, many strategies often described as modern, including transposon mutagenesis and phage-based genome engineering, remain fundamentally empirical in nature, identifying productive mutations without revealing why they work, making it difficult to rationally transfer or build upon the findings^33,141,144^. It is this mechanistic opacity that motivates the shift toward systems biology frameworks, which offer rational and predictive strategies grounded in a mechanistic understanding of the cell. However, high-throughput screening methods remain challenging but can be aided by systems-level approaches (discussed in the next section).

Pathway-specific or targeted activation strategies directly manipulate the regulatory elements embedded within a given BGC. The advent of CRISPR-Cas systems has greatly expanded pathway-specific activation, allowing the installation of strong promoters upstream of biosynthetic genes, modulation of PSTF, or precise editing of regulatory regions to fine-tune individual clusters^32,144,145^. Comprehensive reviews of these activation strategies are available elsewhere^20,31,42^.

Bacteriophages is an emerging source of orthogonal regulatory elements (such as strong promoters, terminators and sigma-like factors) that can be used to refactor silent BGCs in non-model hosts^33^. Phage-derived integrases and recombination systems could enable precise genome engineering, offering new ways for rewiring BGC regulatory architecture.

Although pleiotropic approaches are powerful for uncovering new bioactivities and generating broad experimental data, their effects are diffuse and often unpredictable in magnitude and specificity^31,42,146^. By reshaping the entire regulatory and metabolic state of the cell, they can activate multiple BGCs simultaneously but provide little control over which pathways respond or how strongly they are expressed. This lack of specificity makes it difficult to optimise a single target cluster or translate activation events into stable, high-yielding production^31^.

Pathway-specific approaches offer far greater precision, but depend on detailed knowledge of BGC architecture, regulatory circuitry, and metabolic requirements^31,146^. Predicting how a cluster will behave once activated or transferred into a new host requires understanding its promoter layout, native expression hierarchy, and the metabolic burden it imposes on the cell. This is relatively tractable in well-characterised heterologous hosts, where regulatory and metabolic landscapes are better defined^41^. Even then, challenges arise, including codon-usage mismatches, difficulties in assembling and maintaining large clusters, and metabolic flux imbalances that strain host resources^33,147–150^.

In native or non-model producers, these specific limitations are often less severe because the cluster is already optimised for that cellular background^151^. However, in practice, pathway-specific engineering is even more challenging in these organisms because the underlying biology is far less understood. For many non-model species, basic information about promoter identity, regulatory dependencies, global control systems, and precursor supply routes is limited^151,152^.

Altogether, these challenges highlight why BGC activation is rarely sufficient to achieve meaningful or industrially relevant production. Whether using global or targeted methods, success ultimately depends on aligning regulatory control with the cell’s metabolic capacity. Without this coordination, activation can lead to unstable expression, resource depletion, or rapid selection for non-producing mutants. Realising the full potential of both pleiotropic and pathway-specific strategies therefore requires a deeper understanding of the cellular context in which activation occurs, including how regulatory signals spread, how metabolic resources are redistributed, and why expression so often remains unstable even when clusters are switched on. These insights are increasingly being facilitated by bioinformatic and computational approaches that provide the systems-level resolution needed to navigate this complexity and rationally address the regulatory and metabolic imbalances that limit robust, scalable production.

## Computational modelling approaches for activating biosynthetic gene clusters

Activating a silent BGC and expressing it to its fullest extent is not a single intervention but a sequential problem. After BGC identification, regulatory logic and broader network controlling its expression are mapped, and, finally, the metabolic capacity of the host is assessed. Computational tools have emerged to address each of these steps, and it can be integrated into a unified framework.

Genome mining tools such as antiSMASH have become vital for BGC identification^153^. These tools integrate curated rule-based frameworks with profile hidden Markov models and domain architecture analyses to detect signature biosynthetic enzymes, delineate cluster boundaries, and predict chemical classes. Complementing this rule-based approach, machine learning methods such as DeepBGC^154^, BiGCARP^155^, and BGC-Prophet^156^ leverage data-driven models trained on large genomic datasets to detect clusters that fall outside established BGC rules, capturing the full breadth of biosynthetic diversity that conventional tools may overlook.

Computational tools also aid in the activation of silent BGCs by resolving which TFs govern expression identification of binding sites. The antiSMASH TFBS Finder module addresses this by annotating putative TFBSs within BGC promoter regions, providing direct hypotheses for which regulators control a given cluster^157^. COMMBAT builds on this by addressing the poor sensitivity of standard motif-scanning methods to the degenerate and low-affinity binding sites common in BGC regulatory regions. Combining sequence and functional context into a single integrated score, a more accurate prediction of regulatory binding sites is achieved^158^. The value of this regulatory-first approach is well illustrated experimentally. For example, screening the genome of *Amycolatopsis japonicum* for binding sites of the zinc uptake regulator Zur led to the identification of the *aes* genes responsible for [S,S]-EDDS biosynthesis, a discovery that would have been missed without targeted regulatory analysis^159^. Similarly, integrating regulatory network analysis with co-expression data enabled the functional identification and experimental validation of a previously uncharacterized BGC, highlighting the predictive power of regulatory-guided approaches^77^.

However, resolving individual TF-BGC interactions captures only part of the regulatory picture. Machine learning approaches applied to large transcriptomic datasets are beginning to reconstruct genome-wide regulatory networks across diverse bacterial species. Independent component analysis has revealed independently modulated gene sets (iModulons) that decompose the transcriptome into co-regulated units whose activity shifts quantifiably across growth conditions, allowing researchers to observe how entire regulatory programmes activate or repress BGCs in response to environmental and physiological cues relevant to SM production^160,161^. These data-driven frameworks are bringing us closer to a holistic understanding of how global and pathway-specific signals are integrated to control BGC output, the kind of systems-level insight that cannot be obtained from studying individual regulators in isolation^162^. Therefore, these data-driven regulatory frameworks are not yet intended to replace empirical approaches but to complement them, providing mechanistic context for mutations identified through classical screening and reducing the search space for rational BGC activation strategies.

Finally, a more robust understanding of BGC regulation does not resolve the metabolic burden of SM production on the host. This is a major bottleneck in bioengineering, particularly when using non-model organisms. Genome-scale metabolic models (GEMs) address this by enabling in silico metabolic flux prediction, i.e., flux balance analysis (FBA), that can predict precursor availability, identify metabolic bottlenecks, and guide rational engineering strategies^163,164^. GEMs of *Streptomyces* have been used to model the primary-to-secondary metabolic switch, predicting how cellular resources are redistributed when production is initiated. Comparative FBA across dozens of actinobacterial species has also revealed that organisms encoding the greatest number of BGCs do not necessarily have the metabolic capacity to overproduce their encoded compounds^106^. This means that biosynthetic potential and productive capacity are not equivalent. These insights, unattainable through experimental approaches alone, highlight the predictive power of constraint-based modelling for strain design^163,164^. The integration of multi-omics data with regulatory network models and GEMs, as illustrated in Fig. 1, represents the emerging frontier: AI-assisted frameworks that couple regulatory networks with metabolic modelling offer a powerful route toward the rational activation of silent BGCs and the engineering of robust microbial cell factories. However, current automated approaches for reconstructing BGC-associated metabolic pathways into GEMs remain limited in accuracy and highlight that more sophisticated strain engineering strategies will be necessary^165^.

## Conclusion and outlook

Much of the biosynthetic potential of microbes remains inaccessible because many BGCs are silent under standard laboratory conditions. Overcoming this challenge requires understanding how two tightly connected systems, the regulatory machinery of the cell and its metabolic capacity, work together to determine whether a BGC can be expressed, sustained, and made to produce meaningful yields. BGC activation is a complex process that depends on both regulatory permission and metabolic capability. On the regulatory side, pathway-specific and global TFs, chromatin organisation, and higher-order regulatory networks all influence whether a cluster is expressed. At the same time, achieving robust and scalable production requires sufficient precursor supply, adequate energy availability, translational capacity, and a balance with the demands of primary metabolism and growth.

Current activation strategies often focus on only one of these requirements, whereas a more integrative approach could overcome several limitations of existing BGC activation methods and drive higher, more sustained yields. Combining pleiotropic and pathway-specific experimental strategies with high-throughput multi-omics data, integrated through metabolic modelling and regulatory network analysis, provides a powerful framework for this purpose. Such approaches enable systematic interrogation of regulation-metabolism crosstalk while also delivering mechanistic insight into the behaviour of individual TFs and BGCs. This knowledge supports targeted hypothesis testing that can feed directly into experimental design. By integrating regulatory network models, GEMs, and multi-omics data, can we move from trial-and-error approaches toward robust predictive strategies for unlocking and fully exploiting the biosynthetic potential encoded within microbial genomes.

### Reporting summary

Further information on research design is available in the Nature Portfolio Reporting Summary linked to this article.

## Supplementary information

Reporting summary
